# Oral language skills and mental health in female prisoners: pragmatic skills are essential

**DOI:** 10.3389/fpsyg.2023.1212121

**Published:** 2023-07-31

**Authors:** Frøydis Morken, Wenche Andersen Helland, Elisabeth Evanger, Aslaug Vårvik, Lise Øen Jones

**Affiliations:** ^1^Department of Biological and Medical Psychology, University of Bergen, Bergen, Norway; ^2^Department of Research and Innovation, Helse Fonna Health Authority, Haugesund, Norway; ^3^Independent Practitioner, Bergen, Norway; ^4^Independent Practitioner, Hå, Norway; ^5^Department of Psychosocial Science, University of Bergen, Bergen, Norway

**Keywords:** oral language abilities, pragmatic skills, mental health, anxiety, depression, prison, female prisoners

## Abstract

**Introduction:**

There are strong indications of an elevated incidence of both language problems and problems with mental health among prisoners. We also know that women in prison are a particularly vulnerable group who often face conditions that foremost accommodate the needs of men. In order to adapt prison conditions to women’s needs and give better help, we need more information about their characteristics. In this study, we wanted to explore associations between oral language problems and mental health (depression and anxiety) in women in prison.

**Method:**

Participants were 58 women, recruited from high and low security women’s and mixed prisons. They completed a questionnaire covering demographic variables and several self-report measures. In the present study, the language measures were a Language Composite score (comprising articulation, impressive and expressive language, and pragmatics) and the La Trobe Communication Questionnaire (LCQ), measuring pragmatic skills specifically. Hopkins Symptom Checklist-10 (HSCL) was used as a measure of psychological distress. First, we assessed correlations between the language measures and mental health. Second, we performed group comparisons with groups defined as *over* or *under* cut-off on the Language Composite, LCQ total, and HSCL total scores.

**Results:**

Results supported a clear connection between overall language and overall mental health. Pragmatic skills were the main driver of this effect. There was no difference in mental health between those scoring above and below cut-off for general language problems but the group with possible pragmatic impairment showed poorer mental health than those without. Conversely, there was no difference in general language skills between the groups scoring within and outside the range of psychological distress, but the first group evaluated their pragmatic skills as significantly poorer than the latter.

**Discussion:**

We conclude that pragmatics seem to be core to the association between oral language skills and mental health among female prisoners. This should have implications for language services in prisons, as attending to these issues could positively affect prognosis and outcome.

## 1. Introduction

Even though there are strong indications of an elevated incidence of language problems in prison populations (Bryan, 2004; Bryan et al., 2007; Snow and Powell, 2011; Morken et al., 2021), awareness of language needs is not high in the justice system, and assessment and help are generally not readily available. There is a need for more knowledge about the language problems of people in prison to better understand how language services may benefit this group both prior to, during, and after incarceration. This paper explores associations between language and mental health in female prisoners.

Prisoners are a vulnerable population, and female prisoners particularly so. Women in prison are more likely than men to experience chronic medical disorders, psychiatric disorders, and drug dependence (Binswanger et al., 2010). Many come from very disadvantaged backgrounds of abuse and addiction, while their crimes are most often associated with their life situation and few can be considered a danger to society (Fair, 2009, p. 3). World-wide, the proportion of women and girls in prison is low. Recent numbers estimate the population at 6.9% of the total number of prisoners (3.3% in Africa, 5.9% in Europe, 6.7% in Oceania, 7.2% in Asia, and 8.0% in the Americas) (Fair and Walmsley, 2022, p. 2). Because of the low numbers of women, prisons tend to prioritize the needs and requirements of male prisoners (Fair, 2009). Challenges facing women in prison include older buildings that often cannot offer adequate conditions for physical activity and fresh air, work and spare-time activities that have low priority because of security issues and lack of resources, and health services that are often less available for women than for men. Furthermore, there can be problems related to safety, especially for women serving in mixed prisons (Falkanger, 2016; Jones, 2019). However, even though the proportion of women in prison compared to men is low, there are at any given time more than 740.000 individual women and girls in prison globally (Fair and Walmsley, 2022), underscoring the necessity and importance of addressing the needs of this group. Adapting prison services to the needs of female prisoners is required to assist in their rehabilitation and reentry into society (Bartels and Gafney, 2011; Falkanger, 2016). Still, with a few exceptions, studies of the prison population tend to include mainly male participants. This is perhaps natural, given the proportion of male to female prisoners, but knowing that the characteristics and needs of women in prisons often do not align with those of men it should be evident that more knowledge about these women is necessary. One thing we do know is that even in the general population rates of depression and anxiety are significantly higher among women than men (Cyranowski et al., 2000; Pigott, 2003; Faravelli et al., 2013; Asher et al., 2017), and that in prison an even larger proportion of women suffer from mental health problems (Svendsen et al., 2023).

Mental health can be defined in at least three ways: as absence of disease, as a state that allows the organism the full performance of all its functions, and as a state of balance within the person and between the person and the physical and social environment (Sartorius, 2002). Poor mental health can, in turn, have adverse effects on quality of life (Spitzer et al., 1995; Meule and Voderholzer, 2020) and work-force participation (Frijters et al., 2010), which will also impact possibilities for making an income. Previous research has shown that people with language problems display more symptoms of depression, anxiety, behavioral problems, and difficulties with attention than those without language problems (Beitchman et al., 2001; Conti-Ramsden et al., 2013; Botting et al., 2016; Helland et al., 2022b). Helland et al. (2022b) found that girls with language problems were more likely to experience emotional problems, whereas boys were more likely to display behavioral problems. Moreover, language problems can often be misunderstood by the environment as boredom, evasion, or resistance (Snow and Powell, 2004a; Helland et al., 2014b), and can lead to avoidance of social interaction, aggressiveness, negative behavior, and low self-esteem (Hopkins et al., 2016). For the prison population, Hughes et al. (2017) found that having language needs was associated with a higher risk of self-harm and substance abuse.

In this paper, we use the term *(oral) language problems* to refer to any self-reported difficulties with oral language This means that we do not exclusively refer to persons who qualify for a diagnosis of developmental language disorder (DLD) (Bishop et al., 2017), but rather include a broader range of experienced difficulties in oral language production and comprehension. This is both because opportunities for diagnostic testing were not available at the time and because rates of diagnosis have historically been low. Hence, even though DLD has a population prevalence of around 10% (Norbury et al., 2016), when asking participants through self-report if they have been diagnosed with DLD, it is likely that they have not, even in cases where they do indeed fulfill criteria for a diagnosis. Furthermore, relating to mental health the beliefs a person has about their language ability, and their self-efficacy related to language, may be just as important as results of objective measures. If you feel you have a language problem, that may have a stronger impact than in fact having a language problem and not being aware of it. Self-report may mean both overestimation and underestimation of actual problems (Young et al., 2015), but in our context the most important point is *experienced* problems. Because of the considerations mentioned, we wanted to stay rather wide in scope, and first and foremost focus on experienced problems with oral language.

Having speech and language problems can be difficult not only in terms of general life skills and interpersonal relationships. It can also pose significant challenges for legal protection. Navigating the justice system requires good language comprehension and good expressive skills, for example in forensic interviews and in court proceedings (Snow et al., 2012; Hopkins et al., 2016; Hughes et al., 2017). In an interview study with 31 mainly male young offenders, the majority pointed out that communication abilities were important when presenting in court, and several believed that it could also affect severity of punishment (Hopkins et al., 2016). The bulk of research into these issues, has been done on young offenders, but there is no reason to expect the situation to be significantly different for adults.

Language ability is important from the very first contact with law enforcing authorities where there is often a need to both understand complex language and communicate appropriately (Rost and McGregor, 2012; Snow, 2019). Moreover, there is evidence that listening comprehension is directly related to comprehension of legal rights (Lieser et al., 2019). For example, Lieser et al. (2019) showed that 75% of a group with language disorder did not understand when being read their Miranda rights (the “reading of rights” that is done by police officers in the United States upon arresting someone) compared to 30% in a control group. Furthermore, they did not oversee the consequences of waiving these rights. Naturally, it is difficult to act appropriately if you do not fully comprehend the situation you are in. In the context of court proceedings, Johnston et al. (2016) found that in their sample, many had problems with both core terminology and with understanding court procedures and the roles of the different participants in the proceedings, such as the jury, the witness, the prosecution lawyer, and even the defendant. Notably, there is also evidence that having oral language problems can severely impact prognosis, with young offenders with DLD being more than twice as likely to reoffend as those without language issues (Winstanley et al., 2021). Thus, it is clear that language problems can have great impact on several life arenas, from dealing with the justice system to academic achievement and mental health. Importantly, Hopkins et al. (2016) found that their participants, who were young offenders, were themselves aware of their challenges in communication and literacy. More than half expressed concerns about their language and literacy abilities, and identified clarity of speech, aggression, swearing, and confidence in conversing with others as potential candidates for improvement in the oral domain. This partially reflects that language is a multi-faceted phenomenon which includes several domains and subskills, ranging from the processing and production of the speech-sound signal to complex social communication, and it is possible to have challenges in one or more of these domains. Still, most studies compare impaired and non-impaired groups, without addressing the dimensionality of that impairment. Hence, it is not clear how mental health relates to these different language domains.

However, in a sample of children referred to an outpatient child and adolescent psychiatric clinic, Brenne and Rimehaug (2019) found pragmatic language skills especially to be strongly associated with mental health factors. In their clinical sample, pragmatic skills were lower, and rates of pragmatic language impairment (PLI) higher compared to community samples. Significant correlations were found between pragmatic skills and anxiety/depression, withdrawal, social problems, thought problems, and attention problems. However, they did not find correlations between mental health measures and speech, syntax, social relationships, or special interests. The authors concluded that PLI seems highly comorbid with mental health problems.

As mentioned, most of the research into oral language in prison inmates, has been done on young offenders, with few studies on adults (Morken et al., 2021). Studies generally report a significantly elevated prevalence of speech and language problems compared to the general population. Snow and Powell (2011) used a rather strict criterion to identify language impairment, and 37% of their sample still fell into this category. Bryan (2004) studied different language components in a group of young offenders and found that the number of participants scoring significantly below what is expected for their age differed between language domains (naming 43%, grammatical competency 73%, language comprehension 23%). Other studies found elevated rates of difficulties in prison populations within other language domains, for example figurative and abstract language (Snow and Powell, 2004b), receptive grammar (Bryan et al., 2007), receptive language in general (Hughes et al., 2017), and narrative abilities (Snow and Powell, 2004b). Research has also shown that language impairment and attention deficit hyperactivity disorder (ADHD) often co-occur (Cohen et al., 1998; Tirosh and Cohen, 1998; Sciberras et al., 2014; Helland et al., 2014a, 2016), and that there is increased prevalence of both language problems and ADHD in the prison population (Helland et al., 2022a). It has been suggested that difficulties with pragmatic language skills can explain elevated rates of social difficulties in persons with ADHD (Staikova et al., 2013).

Altogether, this underscores the necessity of exploring the impact speech and language problems can have on prisoners so that prisons can offer the best possible services to assist in rehabilitation. To our knowledge, no previous studies have examined the associations between different language domains and mental health in this population.

### 1.1. Aims

As pointed out, there is a need for raising awareness of language disorders in the justice system, and prisons should seek to provide adequate assessment and intervention (Lount et al., 2018). Hopkins et al. (2016) pointed especially to the need for research on language and communication difficulties in female prisoners. As previously mentioned, research has shown associations between oral language in general, and pragmatic skills in particular, and mental health problems, and there is an elevated prevalence of both language problems and mental health problems in prisons. However, most of this research has assessed men only or mainly. Therefore, the main aim of this study was to explore associations between oral language problems and mental health in a group of female prisoners. We also wanted to investigate the possible link between pragmatic skills and mental health.

## 2. Materials and methods

### 2.1. Participants

Participants were 58 female prisoners who were recruited through the prison education services in Norway. The participants were recruited from four women’s prisons and mixed prisons, and both low and high security prisons were included in the study. Due to missing data, the number of responses to individual questions varied between 53 and 57, except for the question on sentence length which had 46 respondents. All participants were over 18 years old, held a Norwegian citizenship and needed to have sufficient knowledge of the Norwegian language to be able to complete the questionnaire. Demographics on age, sentence length, and education level are presented in Table 1. Due to ethical regulations, response alternatives were given as categories to ensure anonymity in a very limited population. Over time, the rate of female prisoners in Norway has been around 6% (Norwegian Correctional Service, 2022). This corresponds to the rate reported internationally (Fair and Walmsley, 2022), and means that at any given time, there are around 180–200 women in Norwegian prisons. Per July 2021, about 75% of prisoners were Norwegian citizens (Norwegian Correctional Service, 2023). This means the target population was approximately 140 persons, giving an estimated response rate of roughly 40%. Even though recruitment was done via the prison schools, 26 participants (45%) did not take part in education while in prison.

**Table 1:** Demographics.

		Frequency	Percentage
*Age (n = 57)*	18–24	9	15.8
	25–34	14	24.6
	35–44	15	26.3
	45 or older	19	33.3
*Sentence length (n = 46)*	Less than 3 months	11	23.9
	3–6 months	6	13.0
	6–12 months	10	21.8
	1–4 years	10	21.8
	More than 4 years	9	19.5
*Education (n = 57)*	Lower secondary or less	18	31.6
	1^st^ year upper secondary	8	14.0
	2^nd^ year upper secondary	10	17.5
	3^rd^ year upper secondary	11	19.3
	Higher education	10	17.5

Participants were asked to indicate whether they had any difficulties in reading or writing, and they were asked to report whether they had ever – as a child or as an adult – received a formal diagnosis of dyslexia or reading and writing disorder, developmental language disorder (DLD – or specific language impairment, SLI), or ADHD. Results are reported in Tables 2 and 3 respectively. For reasons of anonymity the alternatives yes, as a child and yes, as an adult are collapsed in the presented results in Table 3.

**Table 2:** Self-reported difficulties (*n* = 57).

		Frequency	Percentage
*Reading*	Yes, large difficulties	31	54.4
	Yes, difficulties to some extent	10	17.5
	Yes, some difficulties	12	21.1
	No difficulties	4	7.0
*Writing*	Yes, large difficulties	32	56.1
	Yes, difficulties to some extent	13	22.8
	Yes, some difficulties	8	14.0
	No difficulties	4	7.0

**Table 3:** Formal diagnoses.

		Frequency	Percentage
*Dyslexia (n = 56)*	Yes	19	33.9
	No	37	66.1
*Developmental Language Disorder (n = 57)*	Yes	3	5.3
	No	54	94.7
*ADHD (n = 57)*	Yes	20	35.1
	No	37	64.9

For oral language, participants were asked to assess their skill-level. 12.5% reported weak or very weak skills.

It should be noted that the low number of diagnosed cases of DLD could be a direct consequence of diagnostic practices, since rates of diagnosis have generally been considerably lower than the expected population prevalence of this disorder. This is less true for dyslexia, though we know that even for this disorder many children remain undiagnosed or are diagnosed very late (Solem, 2021).

### 2.2. Procedures

Participants were recruited with the help of the prison administration and the head of the educational services in each prison. Information and consent forms were handed out by the prison teachers, and questionnaires were filled out in class. Participants were allowed to ask questions, and the teachers assisted them when necessary. They were given the time they needed to fill out the questionnaire.

The study was approved by the Data Protection Official for Research, NSD (Norwegian Center for Research Data), and additional approval was granted by the Correctional Services and the prisons taking part in the study. A presentation assessment was also sent to the Regional ethical committee (REK) who concluded that a full assessment was not required.

### 2.3. Measures

The full questionnaire was 11 pages. It included questions on demographics and several different topics, such as incentives and barriers to participation in prison education, motivation and self-efficacy, and insomnia. The scales that were included in the present study are presented below.

#### 2.3.1. Language composite

General oral language function was assessed by self-report on four questions targeting different language domains (phonology, expressive language, language comprehension, and pragmatics): (1) I cannot pronounce certain words or sounds, (2) I cannot elaborate, explain, or express myself, (3) I have difficulty in understanding things that are being said, and (4) I have difficulties having a conversation with others. These questions have been used in previous studies on children, phrased as questions to the teachers and parents, and on adults (Helland et al., 2014b, 2022a,b). Scoring was done on a three-point scale (0 = Not true, 1 = Partly true, 2 = Certainly true). The scores on the four questions were summed to obtain a Language Composite score (range = 0–8), where higher scores indicated more problems. For group analyses, a person who scored at or above 2 on the Language Composite was regarded as having oral language problems. This would correspond to at least two answers of partly true or at least one answer of certainly true. This is in line with the cut-off set by Helland et al. (2022b). Hence, the Language Composite was used as an overall measure of oral language function. Subsequently, results on the individual questions were examined to get an idea of which language domains were driving any observed effects.

#### 2.3.2. La Trobe communication questionnaire

The La Trobe Communication Questionnaire (LCQ; Douglas et al., 2000; Norwegian translation: Hansen et al., 2017) was developed to assess communicative ability in young adults with traumatic brain injury. It consists of two identical questionnaires, to be completed by the primary person and one of their close others. Acceptable internal consistency (>0.90) and test–retest reliability (>0.80) have been reported (Douglas et al., 2007). Helland et al. (2022a) found internal validity to be 0.91 for the Norwegian version used here. In the present study, only self-report was used. The questionnaire consists of 30 questions describing different communicative behaviors. Each item is scored in terms of reported frequency of the behavior (1 = Never or rarely, 2 = Sometimes, 3 = Often, 4 = Usually or always). Higher scores indicate more problems. The questionnaire is constructed to reflect Grice’s (1975) four communication maxims, quantity (being informative), quality (speaking truthfully), relation (being relevant), and manner (being clear) – as well as including supplementary questions related to cognitive aspects and speech rate. We have not conducted our own factor analyses to confirm that the factor structure of the instrument was intact since our sample size was too low to allow for this type of analysis (Mundfrom et al., 2005). A few items require reverse scoring (six in the original, and five in the Norwegian version that was used here). All scores are summed, to obtain an LCQ total score (range = 30–120). Douglas et al. (2000) reported females (n = 88) to be normally distributed around a mean of 50.47 (SD = 9.07) in their normative sample of Australian young healthy adults, whereas (Yggeseth, 2019) reported a mean of 47.64 (SD = 8.46) in a Norwegian sample (N = 361) of healthy adults. In addition to the LCQ total score, we also used the five subscales for further detail.

For the purpose of group analyses, we set a cut-off at 1 SD above the mean as reported by Douglas et al. (2000). Hence, the cut-off was set at 60 points, where values above cut-off were seen as an indication of pragmatic impairment.

#### 2.3.3. Hopkins symptom checklist–10

The Hopkins Symptom Checklist is a measure of psychological distress, with subscales for anxiety and depression. It was developed in the 1950s and exists in several different versions ranging from 5 to 90 items in length. In this study, we used the 10-item version (HSCL), which has been shown to exhibit good psychometric properties, and is recommended for use in both research and clinical settings (Strand et al., 2003; Schmalbach et al., 2021). The items in HSCL have four response alternatives ranging from Not at all (=1) to Extremely (=4). The scores from the 10 items are summed and divided by 10 to calculate a total score (range 1–4). In a Norwegian sample of 9,735 participants between 16 and 97 years old, Strand et al. (2003) found the mean total score for women to be 1.41 (SE 0.007). The cut-off for psychological distress was 1.85 (Strand et al., 2003).

The anxiety subscale (item number 1–4) is calculated based on four questions, and the depression subscale (item number 5–10) is based on six questions. Due to the low sample size, we have not performed our own factor analyses of the HSCL in the present sample but rely on the factor structure presented by Schmalbach et al. (2021).

### 2.4. Data analyses

SPSS version 27 was used for statistical analyses.

First, we assessed associations between oral language skills in general and pragmatic skills in particular with mental health using two-tailed correlation analyses (Pearson’s r). We correlated the Language Composite and the total LCQ score with the total score on HSCL, as well as with the HSCL anxiety and depression subscales. To investigate these relationships in more detail, we also correlated the four questions composing the LC, and the five La Trobe sub-scales, with all three HSCL scales. The non-parametric Spearman’s rho yielded comparable results to Pearson’s r and will not be further reported below.

Second, we performed group analyses with groups defined as over or under cut-off on the Language Composite, LCQ total, and HSCL total scores. None of the groupings violated the assumption of homogeneity of variance, as assessed by Levene’s Test of Equality of Variances. For each measure, a two-tailed Student’s t-test (α ≥ 0.05) was used to compare groups. First, we assessed whether the groups scoring in the range of impairment on the language measures (Language Composite and LCQ) showed significantly poorer mental health (HSCL). Then we investigated whether those scoring in the range of psychological distress on the HSCL, showed significantly poorer language skills (Language Composite and LCQ) than those scoring below cut-off. Cohen’s d was used as a measure of effect size, and interpreted as: 0.20 = small, 0.50 = moderate, and 0.80 = large. Due to the relatively low sample size, group comparisons were also tested non-parametrically using Mann–Whitney U. However, results did not differ considerably from the parametric testing, and hence will not be further reported below.

## 3. Results

### 3.1. Correlation analyses

#### 3.1.1. General language ability and mental health

We found significant correlations between general language skills and mental health overall, as well as with the anxiety subscale. When looking at the different language domains, pragmatics was the only domain with a significant association with overall mental health. Anxiety was significantly correlated with expressive language, impressive language, and pragmatics. There were no significant correlations with articulation, suggesting that any problems with speech-sounds are not central to mental health, but this could also reflect that few women reported difficulties in this area (no answers of certainly true, 14 answers of partly true). Details can be found in Table 4.

**Table 4:** Correlations between general language ability and mental health.

		HSCL total	HSCL anxiety	HSCL depression
Language Composite	*r*	0.31^*^	0.44^**^	0.17
	*p*	0.028	0.001	0.219
	*n*	52	52	52
*Articulation*	*r*	*0.16*	*0.22*	*0.10*
	*p*	*0.234*	*0.111*	*0.448*
	*n*	*55*	*55*	*55*
*Expressive*	*r*	*0.22*	0.36^**^	*0.10*
	*p*	*0.111*	*0.008*	*0.487*
	*n*	*52*	*52*	*52*
*Impressive*	*r*	*0.26*	0.33^*^	*0.17*
	*p*	*0.062*	*0.014*	*0.234*
	*n*	*54*	*54*	*54*
*Pragmatics*	*r*	0.30^*^	0.41^**^	*0.18*
	*p*	*0.028*	*0.002*	*0.199*
	*n*	*54*	*54*	*54*

**Significant at 0.01, *Significant at 0.05.

#### 3.1.2. Pragmatic language ability and mental health

When examining the correlation between pragmatics and mental health more closely, we found that the LCQ confirmed the association with overall mental health and with anxiety. In addition, LCQ correlated significantly with the depression subscale. There were significant correlations between all subscales except quality, which is the Grice maxime requiring truthfulness in communication, and which did not correlate significantly with any of the HSCL subscales. Details can be found in Table 5.

**Table 5:** Correlations between pragmatic language ability and mental health.

		HSCL total	HSCL anxiety	HSCL depression
LCQ Total	*r*	0.55^**^	0.43^**^	0.54^**^
	*p*	0.0001	0.003	0.0001
	*n*	46	46	46
*Quantity*	*r*	0.42^**^	0.41^**^	0.36^*^
	*p*	*0.002*	*0.003*	*0.011*
	*n*	*50*	*50*	*50*
*Quality*	*r*	*0.14*	*0.26*	*0.04*
	*p*	*0.327*	*0.069*	*0.762*
	*n*	*52*	*52*	*52*
*Relevance*	*r*	0.36^**^	0.34^*^	0.32^*^
	*p*	*0.008*	*0.012*	*0.019*
	*n*	*53*	*53*	*53*
*Manner*	*r*	0.46^**^	0.33^*^	0.47^**^
	*p*	*0.001*	*0.021*	*0.001*
	*n*	*50*	*50*	*50*
*Cognitive*	*r*	0.64^**^	0.52^**^	0.63^**^
	*p*	*0.0001*	*0.0001*	*0.0001*
	*n*	*51*	*51*	*51*

**Significant at 0.01, *Significant at 0.05.

### 3.2. Group comparisons

We grouped the sample along several dimensions to assess differences between the groups who scored above (indicating impairment or difficulties) and below (indicating no impairment) threshold on the different parameters.

First, we wanted to investigate whether the persons who fell below cut-off on the language measures, also exhibited poorer mental health than those who fell within the typical range.

To do this, we first compared the HSCL scores of the group who scored above threshold on the Language Composite (n = 13, Mean score HSCL = 2.56, SD = 0.63), showing signs of general language impairment, to those who scored below threshold (n = 39, Mean score HSCL = 2.26, SD = 0.74) and as such showed no or very little sign of impairment. The t-test showed no significant difference between the groups (t = 1.29, *p* = 0.20), and the effect size was small (d = 0.41).

We also looked at pragmatics more specifically, comparing the HSCL scores of the group who scored above 60 on the LCQ (n = 15, Mean score HSCL = 2.61, SD = 0.69), indicating impairment within the pragmatic domain, to those scoring below 60 (n = 31, Mean score HSCL = 2.15, SD = 2.15). This t-test came out significant (t = 2.19, *p* = 0.03), indicating that persons who scored in the range of pragmatic impairment, also reported significantly poorer mental health. The effect size was moderate (d = 0.69).

Second, we wanted to assess whether those scoring above cut-off for mental disorders on HSCL (n = 35) scored differently on general and pragmatic language skills than those scoring below cut-off (n = 11). Results showed no significant differences in general language scores between the two groups. However, the effect size was moderate. For pragmatics, on the other hand, the group who scored in the range for psychological distress, showed significantly poorer results than the group who scored in the non-clinical range, indicating that persons who have poorer mental health, will also often experience problems in the pragmatic language domain. The effect size was large. Details are displayed in Table 6.

**Table 6:** Group comparisons between persons with and without signs of psychological distress according to the HSCL.

Domain	Psychological distress		*t*-value	*p* value	Cohen’s *d*
	Over cut-off Mean (*SD*)	Under cut-off Mean (*SD*)			
General language	1.10 (1.43)	0.42 (0.90)	1.56	0.13	0.51
Pragmatics	57.23 (13.55)	46.36 (10.85)	2.42	0.02*	0.84

Language composite and pragmatic language (LCQ) skills. *Significant at 0.05.

## 4. Discussion

The association between language and mental health is well established (Conti-Ramsden et al., 2013; Botting et al., 2016; Hughes et al., 2017; Helland et al., 2022b), and there are several reports of an elevated incidence of both language problems (Bryan, 2004; Bryan et al., 2007; Snow and Powell, 2011) and mental health problems (Hatton et al., 2006; James and Glaze, 2006) among prisoners. This study sought to explore this association in women in prison, since most previous studies have largely focused on men. Our main findings were that there was a clear connection between overall language and overall mental health. When going into detail, we found pragmatics to be the main driver of this effect. Language production and comprehension showed associations with anxiety only. We also found that though there was no difference in mental health between those scoring above and below cut-off for general language problems, the group with possible pragmatic impairment showed poorer mental health than those without. Conversely, there was no difference in general language skills between the groups scoring within and outside the range of psychological distress, but those with psychological distress evaluated their pragmatic skills as significantly poorer than those scoring outside the clinical range for mental health. This indicates that pragmatics can be central to the association between language and mental health in women in prison.

This is in line with previous findings by Brenne and Rimehaug (2019) who also found pragmatics to have stronger associations with mental health than other language domains, albeit in a different population. Likewise, Cohen et al. (2013) found that as much as 45% of a group of youth referred for assessment and treatment at a mental health center had impairment in higher order language function, compared to 15% of controls. Previously, Snow and Powell (2004a) have pointed to poor social and abstract language skills in young offenders. It is, however, difficult to say which direction the association between these higher order language skills and mental health takes. Most probably the influence is bidirectional. One can speculate that as far as the person is aware of their poor pragmatics skills, this can potentially contribute to insecurity in social situations, which in turn can impact self-esteem and mental health. Notably, since this study is based on self-report, the participants’ self-evaluations are the basis of analysis, meaning that only persons who are indeed aware of their difficulties would show up in the group with suspected impairment. Assessment by close others could complete this picture, but due to ethical regulations this was not possible in this project. On the other hand, mental health problems are known to be associated with being more self-critical (Iancu et al., 2015), meaning that persons with mental health problems possibly evaluate their skills more strictly than those in the typical range.

This resonates with the finding that the subscale of anxiety stood out by showing associations with language production and comprehension in addition to the association with pragmatics. We know that anxiety is negatively correlated with self-esteem and self-efficacy, and positively correlated with dependency and self-criticism (Iancu et al., 2015). Hence, it is conceivable that this effect is rather a manifestation of this group being more unforgiving of themselves and less likely to evaluate their communication abilities positively. In other words, this could be a result of differences in subjective assessment, rather than reflecting objectively poorer skills. This could be clarified by including structured individual language assessment in future studies.

However, direct testing of pragmatic skills is not necessarily straightforward, and the ecological validity of such testing has been questioned (O’Neill, 2007). Pragmatics, or social communication, involves many different subskills, and is extremely context dependent. Direct testing of pragmatic skills often involves either pictures or figurines that are used to depict different scenarios, where the person being tested is asked to somehow interpret and respond to the situation that is shown. This is at best a proxy for real-life social situations, and it is not given that the responses a person gives in a clinical setting would match their behavior in real life. Hence, pragmatic impairments are often more evident in real-life situations than in clinical testing (Botting, 2004). Therefore, it is not uncommon to use questionnaires to close others and/or to the person themselves to assess these skills (O’Neill, 2007; Helland and Møllerhaug, 2020).

When it comes to interventions for adults with pragmatic difficulties, the body of literature is small, and mostly related to groups with either acquired brain injuries or with progressive neurological diseases. In a review of interventions for adults with traumatic brain injury, Finch et al. (2016) found that interventions for social communication skills were generally beneficial. Moreover, they found indications that especially context-sensitive approaches were effective, and more so than the more traditional impairment-specific approach which targets more or less exclusively the damaged function. Context-sensitive approaches, on the other hand, combine impairment-based interventions, functional activities, and context-supported participation in a holistic treatment aimed to improve everyday function (Ylvisaker, 2003). There is great need for more research into interventions for adults with pragmatic impairments without known etiology.

An important side note is that Norwegian prison services are based on the principle of normality, which in short implies that the punishment lies in the restriction of liberty itself. Therefore, people who are imprisoned have the same rights as the rest of the population, and their living conditions in prison should as far as possible resemble those in the rest of society. Furthermore, the whole justice system is explicitly built on humanitarianism, due process of law, and equality of treatment (Department of Justice and Police, 2008). This means that though having your freedom taken away may of course in itself have consequences for your mental health, the effects we see should not be caused by adverse treatment or living conditions. Still, we know that appropriate treatment for mental health problems may not be readily available in prisons, and perhaps even less so than in general society. Moreover, we do not know if the reported psychological distress was present before incarceration, or if it has appeared during the prison stay.

In sum, there seems to be a strong association between especially pragmatic skills and mental health in women in prison, but these are not simple associations, and there is probably reciprocal influence between a number of related factors. This is a situation that is well known in the field of language and related disorders, where a model including risk factors and protective factors is increasingly recognized as a meaningful frame of understanding (Bishop et al., 2017; Compton, 2021). Furthermore, we know that comorbidities are common, which again underscores the high degree of reciprocity in how different personal and environmental characteristics influence each other. This means that there should be an obvious place for professionals with knowledge of these factors in the justice system, e.g., speech and language pathologists and psychologists.

### 4.1. Limitations

This study should be viewed as explorative for several reasons. First, the number of participants is relatively low. On the other hand, the target population is also small, so the response rate was acceptable. The study is also based on self-report only, which, as pointed out by Young et al. (2015), may lead to both underestimation and overestimation. We originally planned for individual testing, but due to the COVID-19 pandemic, we had to abandon this plan. Future studies should preferably combine self-report and individual testing. Finally, we know that a significant proportion of the participants also reported an ADHD diagnosis. In this study, we have not addressed the relationship between ADHD, mental health, and language. However, future studies should seek to shed further light on these associations.

### 4.2. Concluding remarks

This study shows that there is a need for assessment and intervention for language problems among prisoners. Particularly, pragmatic skills should be assessed and addressed through a holistic approach and by explicit discussion of communication strategies and support in developing more appropriate social interaction skills. This could potentially have consequences for the general well-being of women in prison and could also affect prognosis and outcome.

## Data availability statement

The raw data supporting the conclusions of this article will be made available by the authors, without undue reservation.

## Ethics statement

The studies involving human participants were reviewed and approved by Norsk senter for forskningsdata (NSD). The patients/participants provided their written informed consent to participate in this study.

## Author contributions

FM, WH, and LJ contributed to the conception and design of the study. EE and AV performed preliminary literature searches and analyses. FM performed statistical analyses and wrote the first draft of the manuscript. All authors contributed to the article and approved the submitted version.

## Funding

This work was supported by a grant from the County Governor of Vestland, Norway.

## Conflict of interest

The authors declare that the research was conducted in the absence of any commercial or financial relationships that could be construed as a potential conflict of interest.

## Publisher’s note

All claims expressed in this article are solely those of the authors and do not necessarily represent those of their affiliated organizations, or those of the publisher, the editors and the reviewers. Any product that may be evaluated in this article, or claim that may be made by its manufacturer, is not guaranteed or endorsed by the publisher.
